# Energy-Efficient Fall-Detection System Using LoRa and Hybrid Algorithms

**DOI:** 10.3390/biomimetics10050313

**Published:** 2025-05-12

**Authors:** Manny Villa, Eduardo Casilari

**Affiliations:** 1 Programa de Ingeniería Electrónica, Universidad de Investigación y Desarrollo (UDI), Bucaramanga 680001, Colombia; 2 Departamento de Tecnología Electrónica, Telecommunication Research Institute (TELMA), Universidad de Málaga, 29071 Málaga, Spain; ecasilari@uma.es

**Keywords:** fall detection, wearable devices, accelerometer, CNN-LSTM, energy-efficiency, LoRa communication, LPWAN technology

## Abstract

Wearable fall-detection systems have received significant research attention during the last years. Fall detection in wearable devices presents key challenges, particularly in balancing high precision with low power consumption—both of which are essential for the continuous monitoring of older adults and individuals with reduced mobility. This study introduces a hybrid system that integrates a threshold-based model for preliminary detection with a deep learning-based approach that combines a CNN (Convolutional Neural Network) for spatial feature extraction with a LSTM (Long Short-Term Memory) model for temporal pattern recognition, aimed at improving classification accuracy. LoRa technology enables long-range, energy-efficient communication, ensuring real-time monitoring across diverse environments. The wearable device operates in ultra-low-power mode, capturing acceleration data at 20 Hz and transmitting a 4-s window when a predefined threshold in the acceleration magnitude is exceeded. The CNN-LSTM classifier refines event identification, significantly reducing false positives. This design extends operational autonomy to 178 h of continuous monitoring. The experimental and systematic evaluation of the prototype achieved a 96.67% detection rate (sensitivity) for simulated falls and a 100% specificity in classifying conventional Activities of Daily Living as non-falls. These results establish the system as a robust and scalable solution, effectively addressing limitations in power efficiency, connectivity, and detection accuracy while enhancing user safety and quality of life.

## 1. Introduction

Significant increases in global life expectancy in recent decades have accelerated population aging, resulting in a higher proportion of older adults. In fact, by 2050, the number of people over 60 is projected to reach approximately 2.1 billion, representing nearly 22% of the total global population [1]. As a consequence, the incidence of falls has risen, making them one of the leading causes of severe injuries and mortality in this age group. Falls are the second most common cause of unintentional trauma-related deaths globally, with an estimated 684,000 fatalities per year, predominantly affecting individuals aged 60 and older [2,3].

A fall refers to an unintentional event where an individual loses balance and descends to a lower surface, such as the ground or floor [3]. The frequency of falls increases with age, and older adults are at the highest risk of sustaining severe injuries as a result. It is estimated that approximately 28% to 35% of individuals over age 65 experience at least one fall per year, a percentage that rises to nearly 32% to 42% among those aged 70 and older. Such events can result in severe injuries, including fractures in the hip, head trauma, and damage to the upper extremities, frequently necessitating prolonged medical care. Furthermore, falls may trigger post-fall syndrome, characterized by a loss of independence, disorientation, reduced mobility, and depression, ultimately diminishing the quality of life of affected individuals [2].

Approximately 47% of older adults who experience a fall are unable to get up on their own, remaining on the ground for an extended period. This inability can lead to severe physical and psychological consequences, significantly increasing the risk of health complications and mortality [4]. Therefore, early fall detection through remote telemonitoring systems can greatly enhance the quality of healthcare and prevent complications in older adults or individuals with limited mobility. Automatic fall-detection systems (FDSs) enable real-time notifications to caregivers and family members when an incident occurs, ensuring a rapid and timely response. Recent studies have highlighted the importance of telemonitoring in patient health management, allowing continuous and real-time health condition supervision [5]. However, one of the most critical challenges in implementing fall-detection systems is the high energy consumption of wearable devices, which are typically powered by low-capacity batteries. In this context, continuous monitoring without frequent recharging is essential to ensure both efficiency and user acceptance [6] of this type of devices.

Fall-detection solutions are generally classified into three categories: wearable devices, environment-based solutions, and vision-based solutions [7]. Vision- and environment-based systems, collectively referred to as context-aware detection systems, share similar advantages and limitations. Both approaches rely on ambient data capture to monitor and track body movement. Despite advancements in this type of detection, several issues still limit their effectiveness, such as restricted coverage to indoor spaces and privacy concerns associated with camera use [8]. In contrast, wearable fall-detection devices can be seamlessly integrated into clothing or accessories due to their compact size. They are cost-effective and consume less energy compared to context-aware systems. Wearable FDSs typically include microcontrollers, inertial measurement unit (IMU) sensors, and, in some cases, barometers, allowing fall detection based on acceleration, angular velocity, orientation, or altitude changes. Additionally, they are equipped with communication modules that enable near-ubiquitous monitoring and data transmission to remote servers for further analysis [9].

The effectiveness of a FDS depends not only on sensor technology advancements but also on the algorithms responsible for processing data from wearable devices. These computational methods analyze inertial signals to accurately detect falls. Different techniques have been proposed, ranging from simple threshold-based approaches (TBM) to more sophisticated machine learning models, aiming to enhance detection reliability and accurately. TBM approaches, recognized for their low computational demands, can be directly implemented in wearable devices without requiring extensive hardware resources [7,10,11,12,13]. However, their effectiveness is limited when differentiating falls from other high-intensity or sudden movements. In contrast, machine learning algorithms, including deep learning techniques, offer higher classification accuracy but at the cost of increased computational complexity and power consumption [9,13,14,15,16]. To combine the strengths of both approaches, hybrid algorithms have been designed to optimize detection accuracy while improving energy efficiency. These models employ threshold-based detection for initial event identification, benefiting from their simplicity and low energy demand, followed by machine learning techniques to refine the analysis [17]. This integrated approach enables precise and efficient fall detection, reducing false positives and improving system reliability without significantly impacting the device’s battery life [18,19,20,21].

Wearable fall-detection systems often utilize wireless communication technologies with limited range, such as Bluetooth Low Energy (BLE) and ZigBee [22,23]. While these protocols are designed to minimize energy consumption, they require a gateway in close proximity to relay alerts to a monitoring system. In most implementations, this gateway is a smartphone, which must remain in the vicinity of the user at all times, a condition that is not always practical or reliable in many application scenarios. An alternative strategy involves equipping wearables with long-range communication capabilities, such as 3G or 4G connectivity [24]. However, these solutions present remarkable challenges, including increased power consumption that reduces battery life. Additionally, their effectiveness depends on the availability of cellular network coverage, which may not be accessible in all locations. Furthermore, mobile network usage introduces recurring expenses, raising the overall cost of the FDS.

To address these constraints, low-power, long-range communication methods have been recognized as a viable alternative. Technologies classified under Low Power Wide Area Networks (LPWAN), including LoRa, have been investigated for their potential in fall detection due to their ability to transmit data over extended distances while consuming minimal energy. These communication protocols are particularly advantageous for wearable devices that rely on battery power, as they enable continuous data transmission without significantly impacting energy efficiency. Several studies have explored the integration of accelerometers in LPWAN transmitters to improve fall-detection accuracy while optimizing power consumption [9,10,11,12,15,25,26,27,28,29,30]. LPWAN solutions facilitate long-range data transmission with substantially lower energy demands [31], enhancing the operational lifespan of battery-powered wearables. This approach not only improves energy efficiency but also eliminates the dependency on intermediary devices, such as smartphones, ensuring a fluid and reliable operation of the FDS even in environments where Wi-Fi or cellular networks are unavailable.

Furthermore, LPWAN technologies, such as LoRaWAN and Sigfox, use unlicensed frequency bands, minimizing operational expenses and simplifying deployment. These networks offer significant advantages in outdoor environments and areas where conventional communication infrastructure is either unavailable or expensive to implement. Their capability to support thousands of devices and the simplicity of the required hardware significantly reduces both scalability problems and installation and maintenance costs. Conversely, certain cellular-based communication protocols, including NB-IoT and LTE-M, rely on licensed frequency bands [31,32]. These technologies take advantage of existing mobile networks to enhance connectivity, improve security in data transmission, and provide stable long-range communication. This makes them particularly suitable for applications requiring high reliability and seamless integration with established network infrastructures.

This article proposes a fall-detection solution that combines a threshold-based model (for preliminary fall detection) with an advanced deep learning classifier based on CNNs (Convolutional Neural Network) and LSTM (Long Short-Term Memory networks), while leveraging LoRa technology to ensure long-range, energy-efficient communication. The wearable device implements an optimized low-power algorithm, which operates in a power-saving mode, periodically waking up to capture acceleration data at 20 Hz. The detector activates only when a predefined threshold is exceeded, so that a four-second window of acceleration data is sent to a remote server where the analysis with the deep learning model is accomplished to confirm the occurrence of a fall. This design ensures efficient energy usage by minimizing unnecessary transmissions, keeping the LoRa communication module in a dormant state for most of the time. 

Beyond its technical contributions, the proposed system also reflects biomimetic thinking. While it does not directly mimic biological forms, it replicates key functional goals observed in biological organisms—such as self-awareness and adaptive response—within an electronic framework. These capabilities are embodied in the system’s ability to autonomously detect critical events, adapt its behavior based on sensory input, and minimize power consumption by remaining in a dormant state until activation is necessary. 

## 2. Related Work

Effectively and sustainably addressing the challenge of high power consumption in wearable fall detection devices requires approaches that balance energy efficiency with high detection accuracy. This study focuses on two key areas fundamental to the development of our proposed system: fall-detection systems utilizing LPWAN transmission technologies and fall-detection systems employing advanced hybrid algorithms.

### 2.1. LPWAN-Based Fall-Detection Systems

Several studies on health tracking with wearables have leveraged LoRa technology, either directly or through the LoRaWAN protocol, due to its capability for long-range data transmission with low power consumption, which makes this standard particularly suitable for biomedical remote monitoring applications. Patel et al. [25] introduced NXTGeUH, an intelligent middleware platform based on LoRaWAN for real-time vital sign monitoring and fall detection, integrating accelerometers and achieving 96.93% accuracy, 100% sensitivity, and 94.25% specificity. Similarly, Lachtar et al. [28] developed a monitoring system for older adults that transmits positional and fall-detection data via Message Queuing Telemetry Transport (MQTT). Their approach utilized a smart cane embedded with a Teensy 3.2 microcontroller, an LSM303DLHC magnetic sensor, and a GPS module, covering an area of up to 6 km^2^ using LoRa technology. Salah et al. [9] developed a fall-detection system utilizing LoRa for data transmission alongside accelerometer measurements. Their study analyzed different classification approaches, including k-Nearest Neighbors (k-NN), CNN, and LSTM, to evaluate their individual effectiveness in fall detection. The results showed that CNN achieved an accuracy of 95.55%, while LSTM reached 96.78%, demonstrating the potential of advanced computational techniques to improve detection performance.

Beyond LoRa-based approaches, Narrowband IoT (NB-IoT) has also been investi-gated for fall-detection applications. Manatarinat et al. [33] developed a fall detector incorporating accelerometers, gyroscopes, and an NB-IoT interface, which enabled low-power, wide-area communication. This system instantly identified falls by comparing real-time sensor readings against predefined thresholds, automatically notifying up to four emergency contacts via a specific application. The architecture integrated an Arduino UNO R3 board and transmitted data using NB-IoT, formatted as JSON, to the AIS Magellan cloud platform, ensuring efficient real-time emergency response. Cai et al. [16] also explored NB-IoT-enabled fall detection, integrating accelerometers and gyroscopes with a Gradient Boosted Decision Tree (GBDT) algorithm, achieving an 89.2% detection accuracy. Similarly, Wu et al. [13] designed an NB-IoT-powered wearable module incorporating MEMS sensors for fall detection. This device, positioned at the waist, was in charge of recording acceleration and body inclination, transmitting the data for analysis through a threshold-based model with a 90.1% accuracy rate. When processed with a Gated Recurrent Unit (GRU)-based deep learning model, detection performance improved to 92.9% accuracy.

Escriba et al. [34] presented a miniaturized electronic patch designed for continuous monitoring of elderly individuals and dependent persons. This waterproof device automatically detected falls, provided geolocation data, and transmitted alerts via Sigfox low-power network. The system integrated a 3D accelerometer and a GPS module, as well as a 30 mAh battery capable of functioning without recharge during 3 days in low-power mode and for 13 h under continuous GPS tracking. In the performed tests, the system successfully delivered alerts in 67.92% of cases, with partial message reception in 28.31%, and receiving fewer than five messages in only 3.77% of trials, which showed the potentials of the Sigfox network to support efficient and reliable transmission in this type of telemonitoring systems.

FDSs have also benefitted from multi-sensor fusion techniques. The prototype described by Pena Queralta et al. in [15] integrated a LoRa transceiver with accelerometers, gyroscopes, and magnetometers, using edge computing and recurrent neural networks (LSTM) for real-time processing. Their system achieved 91.9% accuracy and 95.3% sensitivity, demonstrating high long-term reliability. Likewise, Liu et al. [14] employed NB-IoT with accelerometers, gyroscopes, and magnetometers, achieving 98.85% accuracy, 98.86% sensitivity, and 99.84% specificity using a CNN-based classifier, which was validated against SisFall and Mobifall public datasets [35,36]. However, some studies using LoRa [28,30] or LoRaWAN [28] with multi-sensor configurations did not report detection accuracy metrics, suggesting that data transmission limitations might require edge computing for preprocessing before transmission.

Threshold-based algorithms have also been widely employed. Huynh et al. [10] implemented a simple threshold-based approach based on accelerometers, gyroscopes, and magnetometers in a LoRa-enabled FDS. Their model achieved 96.3% sensitivity and 96.2% specificity, showing that low-complexity algorithms can still achieve effective fall-detection rates.

One of the most critical challenges in LPWAN-based fall detection is energy efficiency. Salah et al. [9] verified that implementing a CNN model on a LoRa detection unit extended battery life to 53 h, compared to only 38 h when using BLE with the same CNN classifier. Similarly, Escriba et al. [34] highlighted the impact of different operation modes on battery consumption in a Sigfox-based system, reporting a three-day runtime under low-power mode and 13 h under continuous GPS tracking, powered by a 30 mAh battery.

Table 1 provides a comprehensive summary of the reviewed studies, detailing the LPWAN technologies used in fall-detection systems, along with sensor configurations, algorithmic methodologies, detection performance (accuracy, sensitivity, and specificity), and energy consumption (when indicated by the authors), aimed at offering a comparative analysis of the strengths and limitations across different approaches.

Table 1 presents key trends and findings in the use of LPWAN technologies for fall detection. LoRa stands out as the dominant technology, as it is widely adopted due to the combination of long-range transmission capability, suitable throughput, lower deployment costs, and low power consumption. When compared to Sigfox and NB-IoT, for example, LoRa and LoRaWAN, as open standards, present a higher flexibility as users can build their own private network or rely on an external telecommunication operator. Among the applied algorithms, neural network-based models such as CNN and LSTM have exhibited superior effectiveness over other AI methods. Studies using NB-IoT reported 98.85% accuracy and 98.86% sensitivity with CNN, while LSTM achieved 96.78% accuracy and 97.87% sensitivity in LoRa-based implementations [9,14]. Integrating multiple sensors, including accelerometers, gyroscopes, and—in very specific and limited cases—magnetometers, has been explored as a strategy to improve fall-detection accuracy, particularly in studies involving advanced algorithms. In this regard, we must take into account that when compared to other inertial sensors, the accelerometer requires a significantly lower current consumption, making it by far the most widely used sensor for developing prototypes intended to monitor human motor functions [39]. Additionally, the implementation of deep learning models in detection units has significantly extended battery life, reaching over 53 h with LoRa and CNN [9].

### 2.2. Fall Detection Systems with Hybrid Algorithms

Hybrid detection algorithms have emerged as a relatively recent trend in the field of fall detection with wearables. These algorithms combine threshold-based techniques with advanced methods to enhance accuracy, reduce false alarms, and optimize power consumption in the transportable device [40,41,42,43]. This approach optimally distributes computational load and enhances energy efficiency, ensuring that data transmission only occurs when a suspicious event is detected, leaving computationally intensive processes to be executed or ‘outsourced’ in the cloud or on another device with greater computing capacity and fewer battery restrictions than a wearable.

Xu et al. proposed in [19] a two-step approach integrating threshold-based preliminary detection with CNN-based refinement. Their system, which achieved 97.5% accuracy, minimized power consumption by activating detailed analysis only when necessary. The study utilized the WH-NB73 module for NB-IoT-based communication, with the wearable device positioned on the thigh. The so-called Power Saving Mode enabled the device to remain in sleep mode for extended periods. Thus, upon detecting a fall, the WH-NB73 module established a network connection, encoded the fall data as an image, and transferred them to feed a server, which applied a CNN-based analysis to confirm the accident.

Following a similar strategy, Shahzad et al. [20] developed FallDroid, a smartphone-based fall-detection system that utilized an accelerometer and a two-stage classification approach. This method combines TBM with Multi-Kernel Learning Support Vector Machines. The hybrid approach effectively differentiated fall-like events from actual falls, achieving 97.81% accuracy, 99.52% sensitivity, and 95.19% specificity when worn on the waist, and 91.70% accuracy, 95.83% sensitivity, and 88.01% specificity when placed on the thigh. Additionally, FallDroid exhibited a false alarm rate of just one per 59 h of use, displaying high accuracy with low power consumption. Data transmission in this system was performed via cellular networks.

Abbate et al. [21] introduced a smartphone-based fall detection system integrating threshold-based techniques with Artificial Neural Networks (ANNs). The model was trained on 86 fall-like events, including 44 actual falls, achieving 100% classification success. Cross-validation using 10 partitions confirmed these results. In a continuous data-collection campaign with three volunteers and 82 h of monitored movement, no real falls were detected, validating 100% specificity for distinguishing actual falls from irrelevant movements. The system employed built-in smartphone accelerometers alongside an optional external detection unit communicating via Bluetooth.

Yuan et al. [18] implemented a hybrid system integrating threshold-based methods with a finite state machine (FSM) for fall detection. They employed accelerometer-based interruptions to trigger processing only for significant events, optimizing power consumption. The FSM validated these events by analyzing sequential conditions over time to confirm actual falls. This method enabled approximately 78.7 days of battery life using a 200 mAh coin cell battery. Data transmission was facilitated via ZigBee, and the wearable device was positioned on the wrist. The classifier showed 94.97% accuracy in validation tests. Additionally, in many hybrid models, initial threshold-based detection is performed on the edge device, while a subsequent advanced machine learning-based analysis takes place in the cloud [19].

The reviewed studies reveal significant progress in the use of LPWAN technologies for fall detection. However, the challenge of reducing energy consumption without compromising detection capability remains. Based on this review, the design of an energy-efficient architecture is considered, combining threshold-based detection and deep learning models such as CNN-LSTM, along with communication technologies such as LoRa, with the aim of achieving a balance between power efficiency and performance in real-world scenarios.

## 3. Materials and Methods

### 3.1. System Architecture Overview

**Figure 1:**
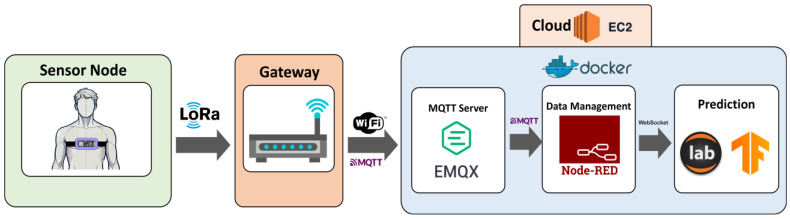
Proposed fall-detection system architecture.

Figure 1 illustrates the proposed system architecture for fall detection. The structure consists of three components: the sensor node, the gateway, and the cloud.

The sensor node is a wearable device that integrates an IMU and a LoRa transceiver to collect and transmit acceleration data. The node employs a threshold-based model (TBM) for a preliminary detection of movements that are suspected to be provoked by a fall. When a potential fall is identified, the node captures two seconds of acceleration data before and after the event. This information is sent to the gateway using LoRa communication technology. The gateway serves as an intermediary element between the sensor node and the cloud layer. Therefore, its main function is to receive data from the sensor node and forward it to the cloud using the MQTT protocol. The cloud layer in turn processes and transmits the data via WebSocket to a hybrid CNN–LSTM model, responsible for confirming fall occurrences. When compared to a policy of constant transmission of the acceleration measurements, the proposed architecture optimizes both energy consumption and bandwidth by performing preliminary detection at the sensor node, limiting cloud transmission to instances where predefined thresholds are exceeded. Thus, only relevant data about ‘suspicious’ movements are transmitted, and the communication module remains in sleep mode when not needed. This approach is also particularly suitable for environments with limited connectivity, as it enables long-range communication with minimal power consumption.

#### 3.1.1. Sensor Node

**Figure 2:**
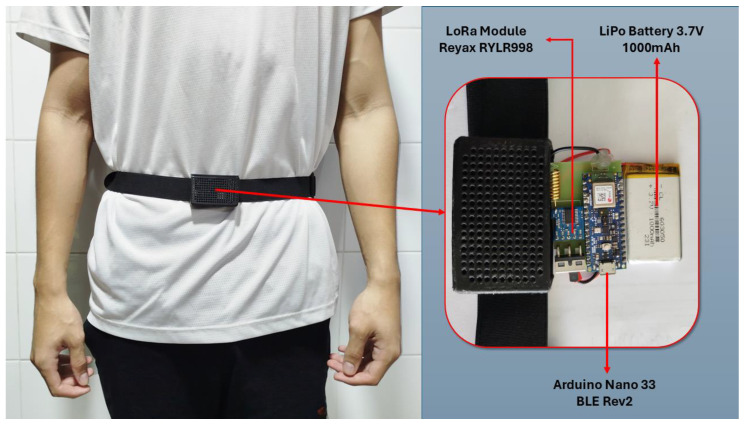
Design and Placement of the Wearable Device.

The sensor node consists of an Arduino Nano 33 BLE Sense Rev 2 microcontroller [44], which acts as the processing core, a triaxial accelerometer (provided by an inertial unit), a LoRa RYLR998 communication module operating at 915 MHz, which enable long-range, low-power data transmission, and a 1000 mAh LiPo (Lithium Polymer) battery allowing for extended operation without frequent recharging. The node was encapsulated in a plastic cover measuring 60 cm (length) × 4.7 cm (width) × 2.4 cm (depth) and specifically fabricated with a 3D printer. Figure 2 illustrates the design and placement of the wearable device, which is attached to the user waist with an elastic band. In this regard, different studies [45,46], have shown that the waist is an optimal election to place an inertial sensor to characterize human movements, as it is located near the body’s center of mass, typically around 10 cm below the navel.

Given that LoRa communication has a limited data-transfer rate [47], the system prioritizes efficient data transmission. To achieve this, only acceleration data is transmitted, excluding other inertial signals that would increase energy consumption and data volume without necessarily improving the effectiveness of the detection decision (see the work in [48] for a further discussion on this topic). Additionally, instead of transmitting the three separate acceleration components, the sensor node computes and transmits only the acceleration magnitude. This approach reduces data size and transmission delay, while ensuring that information reaches the gateway efficiently without compromising speed or device autonomy.

For that purpose, the sensor node continuously calculates the magnitude of acceleration [49] in real time using Equation (1):

(1) $$\left|\vec{a}\right|=\sqrt{a_{x}^{2}+a_{y}^{2}+a_{z}^{2}}$$

where $a_{x},a_{y},\;\mathrm{and}\;a_{z}$ represent the acceleration components along each axis [48], gathered for a particular measurement. In order to maintain a recent history of motion data, the computed magnitude is stored in a 2-s circular buffer at a sampling rate of 20 Hz. The power of the human movement spectrogram is primarily concentrated in frequencies below 10 Hz [50,51,52]. Consequently, we selected this sampling frequency of 20 Hz as a trade-off to simultaneously achieve a proper characterization of human mobility and to minimize computation, memory, and energy costs due to the management and transmission of unnecessary data.

**Figure 3:**
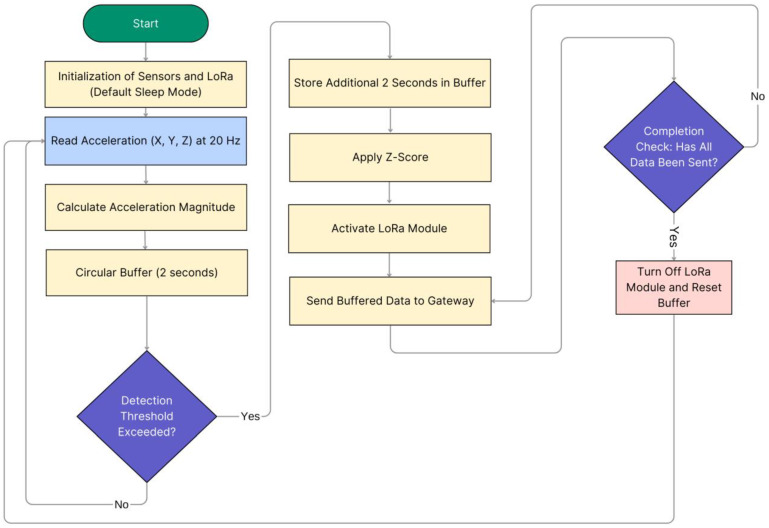
Sensor Node Algorithm for Fall Detection and LoRa Transmission.

Falls provoke a sudden peak or local maximum of the acceleration magnitude caused by the impact of the body against the floor. Thus, the system focuses the analysis on the evolution of the acceleration around these peaks. Ibrahim & Younis have investigated in [53], the acceleration peaks measured at the hip, chest, and head during different types of falls and conventional routines and concluded that falls are always associated with acceleration peaks higher than 3 g (or 29.43 m/s^2^). Thus, in our detector, we take this value as a reference to presume the possible occurrence of a fall. Very similar values have been selected in other proposals of threshold-based FDSs in the literature [53,54,55]. Besides, a typical fall event lasts between 2 and 4 s [56]; therefore, a 4-s observation window around the acceleration peak was selected to ensure a complete capture of the event [55]. Accordingly, in our wearable device, whenever the acceleration magnitude surpasses a predefined threshold of 3 g, the node stores an additional window of 2 s of acceleration measurements in the buffer, gathering a total of 4 s of event-related data. When the 4-s window is obtained, the LoRa module, which remains in sleep mode by default, is activated to transmit the 4-s window (80 samples) to the gateway. Once the transmission is complete, the LoRa module returns to sleep mode, and the buffer resets, resuming normal monitoring operations. This simple pre-detection algorithm, summarized in Figure 3, ensures an efficient use of radio transmissions while optimizing power consumption.

#### 3.1.2. IoT Gateway for Data Transmission

The gateway, implemented with an ESP32 microcontroller and an RYLR998 LoRa module, receives the 80 acceleration samples corresponding to the 4 s window generated by the sensor node upon detecting a potential fall. The ESP32, connected to a Wi-Fi network, utilizes the MQTT protocol [57] to transmit these data to the EMQX broker hosted on an AWS EC2 instance.

#### 3.1.3. Cloud Processing Layer for Fall Detection

**Figure 4:**
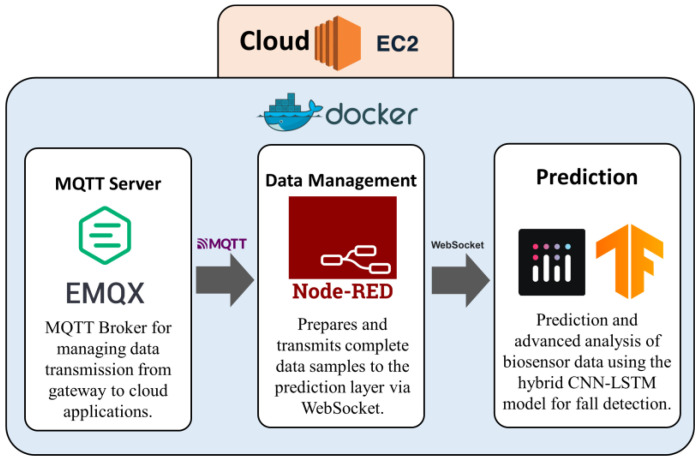
Cloud Processing Layer Architecture for Fall Detection.

The cloud processing layer in the fall-detection system consists of three main components, all deployed in Docker containers on an Amazon Web Services (AWS) EC2 instance: an EMQX MQTT broker [57,58,59], an online Node-RED programmed module, and a Python-based application for movement classification [60]. Figure 4 illustrates the basic interaction flow between these components.

- MQTT EMQX Broker: Receives the 80 acceleration samples corresponding to the 4-s window transmitted from the gateway through MQTT messages.
- Node-RED Container: operates as an intermediary for data management. Through a MQTT subscription to the EMQX broker, the Node-RED container receives the acceleration samples, verifies data integrity, and certifies that the samples are complete and formatted correctly. If missing data is detected, Node-RED applies an interpolation mechanism using the last valid sample to maintain a complete set of 80 samples.
- Movement classifier: Once Node-RED processes and organizes the data, the samples are transmitted via WebSocket to a Python application developed using Dash [61], a framework for building interactive interfaces [61]. The input data undergo Z-score normalization, referencing statistical metrics (mean and standard deviation) derived from the dataset used to train the model. A hybrid CNN-LSTM model then analyzes the data in real time, identifying patterns associated with potential fall events. In addition, in the current status of our prototype, an online application provides a dynamic and user-friendly visualization of both the input data and classification results.

### 3.2. Cloud Detection System

**Figure 5:**
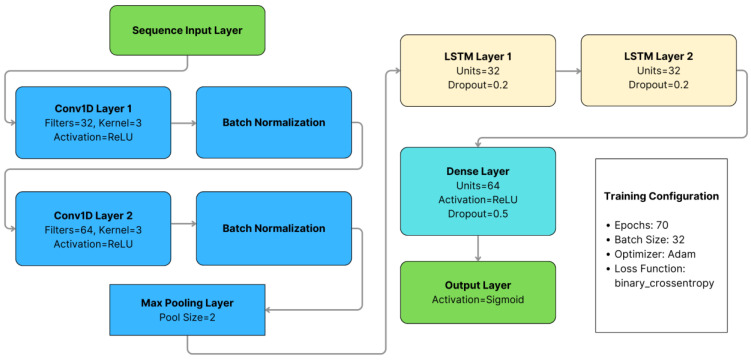
Structure of the Proposed CNN–LSTM Architecture.

The implemented CNN–LSTM model for fall detection (sketched in Figure 5) incorporates a feature extraction module and a sequence modeling unit, leveraging the strengths of both techniques for temporal data analysis. The model’s hyperparameters were selected through a hybrid strategy. Initially, a structured grid search was conducted to explore various combinations of batch sizes, convolutional filter sets, training epochs, and LSTM units, establishing a performance baseline. Based on the most promising configurations, additional manual adjustments were introduced to enhance training stability and mitigate overfitting. This process led to the final architecture—batch size of 32, 70 training epochs, filters of (32, 64), and two LSTM layers with 32 units each—which achieved an accuracy of 98.51% on the test set. This approach is consistent with recent findings suggesting that targeted manual tuning, following exploratory searches, can be more effective than purely exhaustive strategies for hyperparameter optimization [62].

The first module, responsible for feature extraction, consists of two one-dimensional convolutional layers (Conv1D) with 32 and 64 filters, respectively, each utilizing a kernel size of 3. These convolutional layers apply the ReLU activation function to introduce non-linearity, followed by batch normalization to improve stability and accelerate training. Additionally, a max-pooling layer with a window size of 2 is included to decrease dimensionality while preserving the most relevant features.

The feature extraction module identifies spatial patterns within acceleration sequences, facilitating the detection of fall-related characteristics. The output of each convolutional layer can be mathematically expressed as:

(2) $$y_{i}=f\left(\sum_{k=1}^{K}x_{i+k-1}\cdot w_{k}+b\right)$$

where $y_{i}$ represents the output value at position *i* in the feature map. The activation function $f\left(x\right)=\max\left(0,x\right)$ corresponds to the ReLU function, introducing non-linearity. The variable *x* denotes the input data, while $w_{k}$ represents the filter weights of size *K* and *b* is the bias applied during the convolution operation [63].

The second module, responsible for sequence modeling, consists of two LSTM layers with 32 units each and a dropout rate of 20% to prevent overfitting. This component captures temporal dependencies in the data, which is crucial for interpreting the dynamics of a fall. Each LSTM layer processes input sequences based on the operations outlined in Table 2 [64].

A fully connected layer equipped with a sigmoid activation function is employed to differentiate between fall and non-fall events. This layer processes the model’s output, mapping it to a probability score between 0 and 1, which indicates the estimated likelihood of a fall occurrence. To boost the training process, the Adam optimizer is utilized over 70 epochs with a batch size of 32. Additionally, the binary cross-entropy loss function is implemented, as it is particularly effective in refining the accuracy of binary classification models.

### 3.3. Data Preparation and Preprocessing

For the development and training of the CNN-LSTM model, data from 10 subjects, all aged between 19 and 30 years, were selected from the well-known SisFall dataset [35]. This repository is widely used in fall-detection research due to the extensive typology and number of fall events and conventional activities or ADLs (Activities of Daily Living) that it encompasses. In particular, this dataset includes the data captured by two accelerometers and a gyroscope located on the subjects’ waist during the execution of predefined movements (19 types of ADLs and 15 types of simulated falls). In the repository, an individual comma-separated-value (CSV) file -or trace- is provided by the authors for each monitored activity.

**Figure 6:**
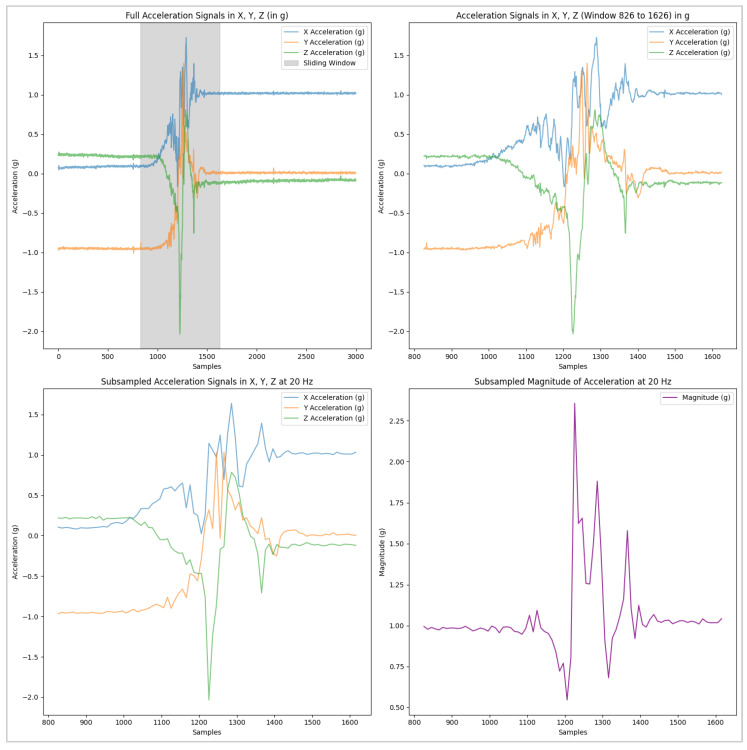
Segmentation and downsampling of the acceleration signals in the original datasets to obtain the final 20 Hz patterns used for training and testing the model.

To train our model, the traces in the dataset (both from samples of falls and ADLs) were processed as follows:

- Data selection: Only the first three columns of each file were used, corresponding to acceleration measurements from an ADXL345 accelerometer on the X, Y, and Z axes, with an original sampling frequency of 200 Hz. Additional columns containing rotation data and readings from the other accelerometer were excluded since our fall-detection approach exclusively relies on the acceleration magnitude computed from a single accelerometer.
- Conversion to gravity units (g): The ADXL345 sensor data requires conversion to gravity units (g) for accurate interpretation. The transformation follows the equation based on sensor specifications (13-bit resolution and ±16 g range):
  (9) $$Acceleration\;\left(g\right)=\left(\frac{2\times range}{2^{resolution}}\right)\times Data\;in\;bits$$
  where the range is ±16 g and the resolution is 13 bits. This conversion standardizes acceleration values across the X, Y, and Z axes for better interpretability and subsequent analysis.
- Segmentation into temporal windows: The duration of the different traces in the Sisfall dataset is extremely variable, ranging from 10 to 180 s. Therefore, for model training, validation, and testing, different 4-s windows (80 samples at 20 Hz) were obtained from the original data. In particular, for each fall, we extracted a single observation window centered around the instant where the acceleration maximum is computed as the rest of the trace is considered not particularly relevant to characterize the dynamics of the fall. As for the ADLs, we selected from each trace at least three 4-s windows that exhibited acceleration peaks likely to be confused with those caused by falls. For this operation, a visual inspection of the signals was combined with the search for the local maxima of the acceleration components. In addition, a downsampling from 200 Hz to 20 Hz was applied to the time series of the patterns to ensure compatibility with the sampling rate of the accelerometer in our prototype. Figure 6 illustrates an example of this process of selection and subsampling accomplished to obtain the final observation windows from one original fall trace in the Sisfall dataset. In the final step (after subsampling), the acceleration magnitude is computed from the acceleration components.
- Z-score standardization: To standardize the measurements and improve model generalization capability by reducing the impact of individual variations in subject acceleration [65], the Z-score normalization was applied to the acceleration data. The formula used is:
  (10) $$Z=\frac{X-\mu }{\sigma }$$
  where each sample within the window has an acceleration magnitude represented by X while μ and σ correspond to the mean and standard deviation, respectively, calculated from the training dataset [63].
- Data Partitioning: To provide a balanced distribution between fall events and daily activities, the dataset was split into three subsets. The division was performed guaranteeing that all subsets include samples of the 19 daily activities and 15 fall simulations executed by the 10 subjects. The training set accounts for 60% of the data, incorporating information from six subjects. Following a leave-2-out evaluation, the validation set comprises 20% of the data, sourced from two subjects not included in the training set, while the test set also includes 20%, using the measurements gathered from two additional different participants. This partitioning ensures a well-represented dataset, enhancing both training reliability and model evaluation [9].

### 3.4. Evaluation Metrics

To evaluate the CNN–LSTM model’s effectiveness in fall detection, several classification metrics were calculated to thoroughly evaluate the model’s effectiveness in differentiating fall events from non-fall cases. The assessment includes accuracy, which represents the percentage of correctly classified instances compared to all predictions made; sensitivity, which evaluates how effectively the model detects actual falls; and specificity, which measures the precision in identifying non-fall cases. These metrics, which are massively employed in the evaluation of binary decision systems (in our case: fall or non-fall), are obtained from the confusion matrix. They basically describe the proportion of correctly identified falls, accurately classified non-fall events, misclassified non-fall instances, and undetected falls within the test dataset [66]. These quality metrics are formally defined as follows:

(11) $$Accuracy=\frac{TP+TN}{TP+TN+FP+FN}$$

(12) $$Sensitivity=\frac{TP}{TP+FN}$$

(13) $$Specificity=\frac{TN}{TN+FP}$$

In these formulas, TP represents the number of correctly identified fall events (True Positives), TN corresponds to the number of ADLs properly identified by the model as non-fall cases (True Negatives), FP indicates the number of non-fall instances mistakenly classified as falls (False Positives), while FN refers to actual falls that were not detected (False Negatives). Each of these metrics contributes to a comprehensive evaluation of the model’s effectiveness, providing a precise measurement of its capability to detect falls while reducing false alarms [67].

## 4. Results

### 4.1. Evaluation of Model Performance

To assess the advantages of the election of our deep learning classifier, a previous comparison is conducted among six different machine learning and deep learning strategies, commonly considered by the related literature: two K-NN (Nearest Neighbors) models (with five and 15 neighbors), a SVM (Support Vector Machine) algorithm, a CNN, an LSTM with batch normalization, and the primary reference model (CNN–LSTM). The objective of this comparison is to evaluate the accuracy, sensitivity, and specificity of each model in fall detection to determine their effectiveness compared to the CNN–LSTM model. The training and evaluation are performed on Google Colab using an NVIDIA Tesla T4 GPU, leveraging its processing capabilities to optimize performance for each tested model. The performance of each model, which are trained and tested with patterns derived from the Sisfall dataset, are summarized in Table 3 [9,35].

**Figure 7:**
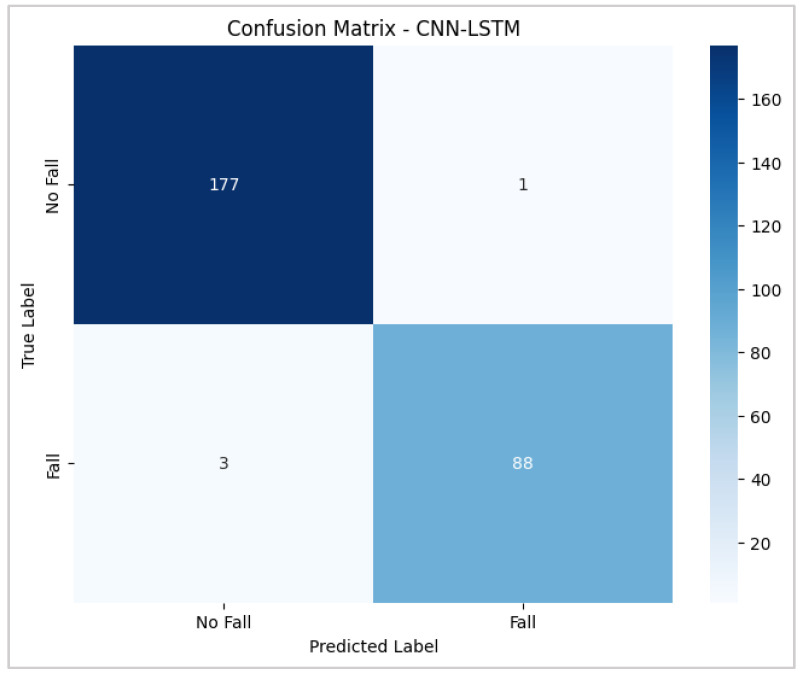
Performance Evaluation Matrix for the CNN–LSTM Model.

As reported in Table 3, the CNN–LSTM model achieved outstanding performance, simultaneously yielding a higher accuracy, sensitivity, and specificity than those achieved by the other classifiers. These findings emphasize the model’s ability to accurately detect fall events while minimizing incorrect alerts, making it a reliable candidate for deployment in wearable fall-monitoring systems. Figure 7 depicts the confusion matrix obtained by the CNN–LSTM classifier, showing the model’s proficiency in differentiating between fall and non-fall events as just four patterns (one derived from a fall and three from ADLs) in the test set were misclassified. The low frequency of false positives and false negatives further validates its effectiveness in correctly distinguishing actual falls from daily routine movements, a critical feature for real-time continuous monitoring systems.

**Figure 8:**
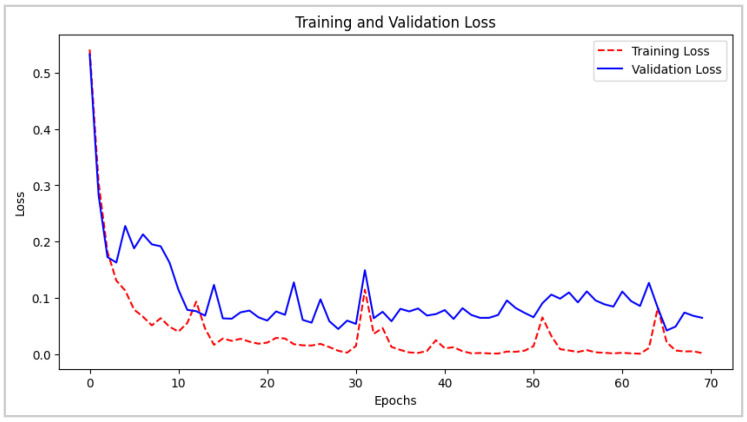
CNN–LSTM Architecture: Training and Validation Loss Evolution.

**Figure 9:**
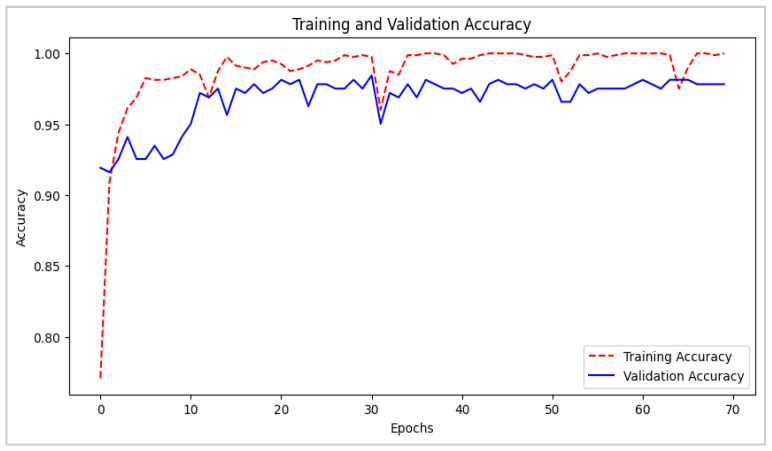
CNN–LSTM Architecture: Training and Validation Accuracy Metrics.

Additionally, Figure 8 and Figure 9 depict the evolution of loss and accuracy over the course of 70 training and validation epochs. In Figure 8, a gradual decrease in loss is evident in both phases, reaching stable and consistently low values towards the final stages of training. This suggests that the model efficiently minimizes classification errors. Figure 9 represents the accuracy trends across both training and validation sets, evincing a high degree of alignment, with values approaching 100% and no apparent signs of overfitting. These results confirm the model’s adaptability and generalization capability, ensuring precise and reliable predictions when applied to new data in real-world environments.

### 4.2. Energy Consumption

Efficient energy management is a key factor in the design of wearable devices for fall detection, particularly in continuous monitoring applications. To assess energy consumption, detailed measurements of the current drain were conducted using a Fluke 87V multimeter (Fluke Corporation, Everett, WA, USA) [68] and analyzing four operating states: microcontroller active with the LoRa module active, microcontroller active with the LoRa module suspended, microcontroller in sleep mode with the LoRa module suspended, and consumption during data transmission. During the test, in order to minimize the impact of other components in the prototype, the power LED of the Arduino Nano 33 BLE Rev 2 board (Arduino S.r.l., Monza, Italy) was deactivated. The obtained results are presented in Table 4.

The device uses a 1000 mAh battery, with an operational safety margin of 80% (800 mAh) to preserve its lifespan. In the selected operating state (microcontroller in sleep mode with LoRa module suspended), energy consumption is 4.5 mA, providing an approximate autonomy of 178 h. This result represents an 81.5% reduction compared to the state where both components are active (24.3 mA). During this state, the microcontroller wakes up only to capture data at 20 Hz, optimizing energy consumption. In contrast, during data transmission, energy consumption increases to 56.2 mA, as both the microcontroller and the LoRa module operate in fully active mode. Although this state exhibits the highest consumption, its impact on autonomy is limited, as each transmission requires approximately 12 s to send 4-s data windows, considering three retransmissions per packet.

### 4.3. Communication via LoRa

As aforementioned, LoRa technology was selected as the communication protocol for the system due to its ability to transmit data over long distances with low energy consumption, which are essential features for environments with limited connectivity. Comprehensive tests were carried out across multiple environments (including different indoor communications within university facilities, as well as in urban and suburban scenarios) aimed at analyzing transmission performance. As quality metrics we estimated parameters such as maximum communication range, data integrity (characterized in terms of Packet Reception Rate or PRR), signal quality (SNR or Signal-to-Noise Ratio), and reception strength (RSSI or received signal strength indicator). Table 5 provides an overview of the obtained results for the different test scenarios.

In indoor environments without obstacles, the system achieved point-to-point transmissions of at least 100 m (in a corridor of a university building) with 100% data integrity, maintaining a noteworthy power level and signal quality at the receptor (RSSI of −49 dBm and an SNR of 14 dB). In the presence of walls, the range was reduced to 50 m, data integrity dropped to 95%, and signal quality decreased with an RSSI of −88 dBm and an SNR of 8 dB. In urban environments, interference from buildings and trees affected performance, reducing reception to 87% at 750 m, with an RSSI of −91 dBm and an SNR of −6 dB. These results confirm the suitability of LoRa for indoor and urban applications, ensuring efficient and reliable communication. Additionally, optimized data transmission following event detection contributes to the system’s energy efficiency.

### 4.4. Real-Time Testing of the Detector in a Testbed

**Figure 10:**
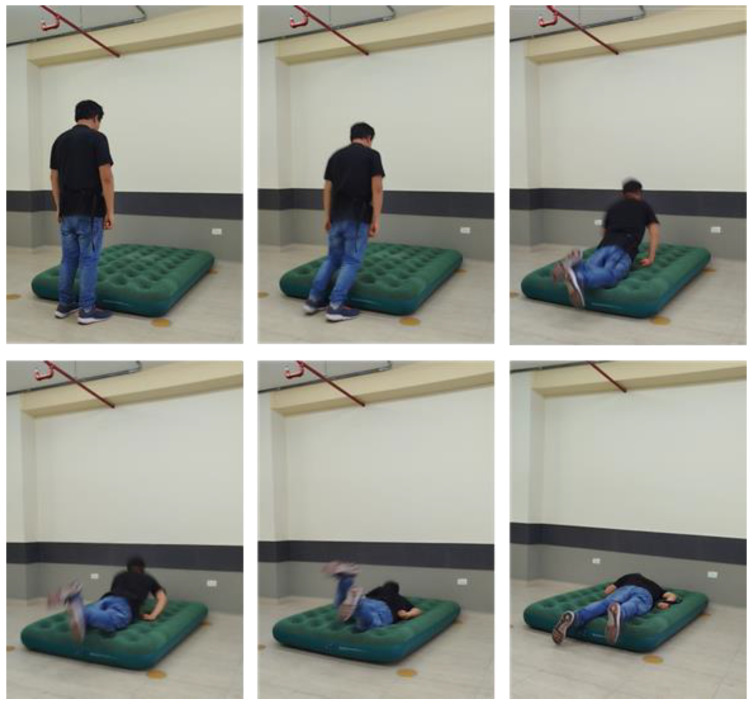
Sequence of frames captured during a forward fall ending in a lying position.

In a second phase, we evaluated the real-time operating capability of the developed prototype by subjecting it to various simulated falls. For this, five volunteers (two women and three men), aged between 20 and 35, were recruited. They performed 60 falls in four different directions and 60 movements corresponding to four basic conventional activities (lying down and getting up, standing up, sitting in a chair and standing up again, and walking and bending down). During the tests, as already mentioned, the participants transported the sensing device on the waist. All falls were performed on a 5 cm thick mat to ensure the safety of the participants, who executed movements following precise and standardized instructions. To illustrate the dynamic nature of the test falls, Figure 10 presents a sequence of frames captured during a forward fall ending in a lying position.

**Figure 11:**
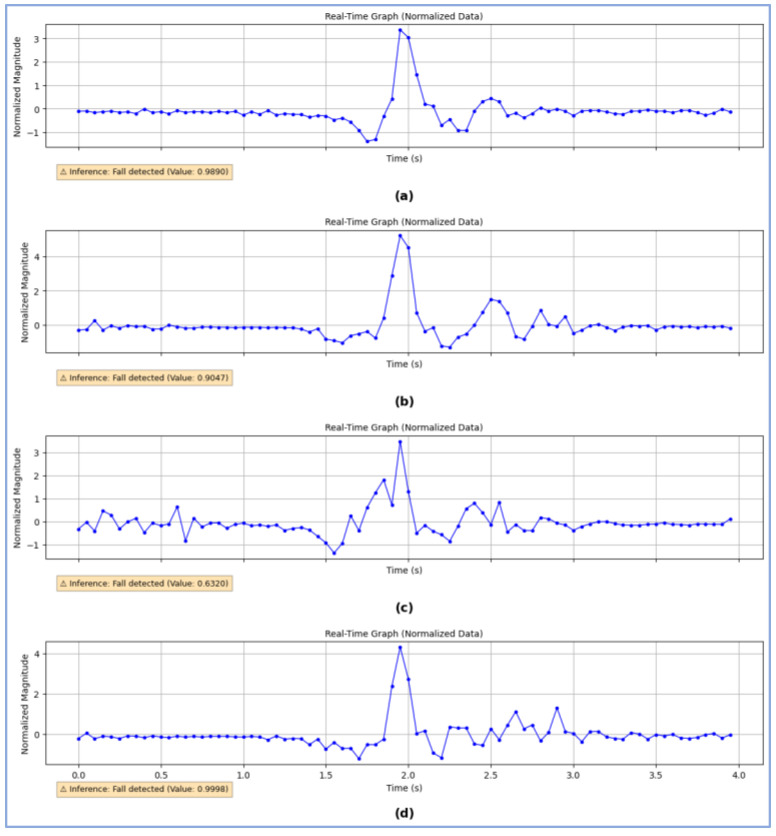
Input (acceleration data) and output (inference value, represented under the curves) of the CNN–LSTM model for different real-time experiments against simulated falls: (**a**) Forward fall ending in a lying position, (**b**) Lateral fall, (**c**) Backward fall with rotation, ending face-down, (**d**) Backward fall ending in a supine position.

On the other hand, Figure 11 presents four samples of the graph of the data collected by the device and sent to the detection application running the CNN-LSTM model after exceeding the threshold during the execution of four different falls. The inference value (or final output) generated by the CNN-LSTM model after processing each data series is also displayed. These graphs highlight the characteristic patterns of each evaluated movement, allowing differentiation between fall events and conventional activities.

During the conducted tests, the system demonstrated high performance in detecting simulated falls, successfully identifying most of the evaluated events. A detailed summary of these test results is shown in Table 6.

On average, the system correctly detected 96.67% of falls, exhibiting great effectiveness in both the threshold-based detection phase and the advanced classification stage of the model. Besides, Table 7 presents the system’s results in differentiating ADLs from falls. The evaluation assessed the threshold’s efficiency in ignoring non-critical activities and the CNN–LSTM model’s capacity to reject those ADLs that exceeded the threshold. As it can be observed, just four ADLs (out of 60 movements) were misinterpreted as ‘candidate’ falls by the thresholding method, but then they were correctly identified as ADLs by the deep learning mode. These results highlight the system’s ability to minimize false alarms and ensure reliability in real-life scenarios.

### 4.5. Real-World 24-h Monitoring

To validate the system’s specificity in a real-world, uncontrolled environment, a 24-h continuous monitoring test (from 8 a.m. to 8 a.m.) on the following day was conducted in which an adult subject (aged 41) wore the wearable device during their daily routine. The ADLs performed during that 24-h period included walking, sitting and standing up, lying down and getting up from bed, ascending and descending stairs, sleeping, cooking, and working at a desk. Throughout the entire test period, no falls occurred and no false positives were recorded. Notably, the acceleration threshold was never exceeded, meaning that no data were transmitted and the CNN-LSTM classifier was not triggered. Therefore, the observed 100% specificity is solely attributable to the threshold-based detection method implemented at the sensor node, demonstrating its effectiveness in accurately distinguishing between routine activities and fall events under realistic usage conditions.

### 4.6. System Cost Breakdown

To evaluate the feasibility of real-world deployment, a cost analysis was performed. Table 8 summarizes the expenses associated with both the wearable device and its gateway, including cloud infrastructure. The cost of the internet data plan required for gateway connectivity is not included, as it may vary significantly depending on the deployment environment, available network infrastructure, and service provider.

## 5. Discussion

The proposed system, which combines a threshold-based model with a CNN–LSTM algorithm and LoRa communication, represents a significant advancement in accurate fall detection with low power consumption, making real-world implementation feasible. The results show that the CNN–LSTM model achieved an accuracy of 98.51%, a sensitivity of 96.70%, and a specificity of 99.44%, outperforming traditional fall-detection methods [10,11,25,26,27,28,33]. In comparison, previous similar approaches in the state-of-art, such as combining LoRa with CNN, achieved 95.55% accuracy in the work by Salah et al. [9], while using LSTM classifiers with LoRa reached 91.90% accuracy in the tests reported by Pena Queralta et al. in [15]. We think that this improvement is due to the integration of the threshold-based model with a deep neural network, allowing effective differentiation between ADLs and potential falls (e.g., sitting, bending, or walking) before applying a detailed evaluation with CNN–LSTM.

The performance of the developed model aligns with previous studies demonstrating the benefits of hybrid approaches in fall detection. In 2019, Xu et al. [56] developed a CNN–LSTM model with a 50 Hz sampling rate, achieving 98.98% accuracy. In 2021, they proposed a two-step CNN method, reaching 97.02% accuracy [19]. Although both models used a higher sampling frequency, our hybrid model, based on thresholds and CNN–LSTM, achieved 98.51% accuracy with only 20 Hz, optimizing energy consumption and reducing the amount of data transmitted via LoRa. Furthermore, the analysis of false positives and false negatives confirms the reliability of the approach, achieving 100% accuracy in classifying non-fall activities after the initial selection stage, reducing false alarms without affecting sensitivity.

The system’s autonomy of 178 h with a consumption of 4.5 mA in sleep mode within a LoRa- and CNN–LSTM-based framework represents a significant improvement over other approaches. Comparatively, Salah et al. [9] reported 53 h with a 2000 mAh battery, while the autonomy of the prototype reported by Zanaj et al. in [29] reaches between 23 and 36 h with 500–800 mAh batteries.

In our system, the energy efficiency is attributed to three key factors. Firstly, the selective transmission of data, by sending only 4-s windows with 80 samples after detecting a potential fall, minimizes LoRa activation and reduces power consumption during idle periods. Secondly, the hardware optimization strategies, such as disabling non-essential components (e.g., LEDs) and using the sleep mode of the Arduino Nano 33 BLE, also contribute to reducing energy expenditure. Finally, the microcontroller remains in low-power mode and only activates briefly to capture data at 20 Hz before returning to sleep, significantly reducing power consumption without compromising system performance.

Signal quality in different environments is affected by the presence of obstacles. In indoor environments with line-of-sight (LOS), packet reception reached 100% at a distance of 15 m. However, when passing through five hollow brick walls, the reception rate decreased to 95%, and in the presence of a concrete slab, it was reduced to 98.75%. In a suburban LOS environment, packet reception reached 100% at 360 m, using a Spreading Factor (SF) of 7, with an SNR of −2 dB and an RSSI of −82 dBm, employing a gateway positioned 40 m high. In contrast, in an urban environment, the signal experienced greater degradation due to the presence of buildings and trees, achieving 87% reception at 750 m. These results are consistent with the findings of Zanaj et al. [29], who obtained a reception rate of 95% at 256 m with LOS, validating the system’s reliability in real-world scenarios. In comparison with NB-IoT, which offers performance similar to LoRa but with higher power consumption [69], LoRa remains an efficient and robust alternative for applications in environments with different levels of obstruction. The proposed LoRa-based system showed reliable performance in obstructed indoor environments. In contrast, Verma et al. [70] noted that BLE’s single-hop design limits its reliability in such settings. Fernandez-Bermejo et al. [41] emphasized that BLE-based systems are often restricted to a 20-m range and therefore require the users to constantly carry a smartphone, which is impractical in most indoors application scenarios. Gharghan et al. [71], also observed signal degradation in ZigBee under NLOS indoor conditions. These limitations highlight the advantages of the proposed system for real-world deployment.

The developed system maintains consistency with the SisFall dataset [35], in which data were collected using the ADXL345 sensor and the unit was placed at the waist. For real-time testing, a wearable device based on the BMI270 sensor (Bosch Sensortec GmbH, Kusterdingen, Germany) was used, introducing possible biases due to differences in data acquisition. However, normalization using the Z-score helped mitigate these effects, allowing the model to maintain high performance. The results were satisfactory, achieving an average of 96.67% accuracy in detecting simulated falls and 100.00% accuracy in classifying daily activities as non-falls in real time. Indeed, the final evaluation carried out has followed a cross-dataset approach, which is generally not considered in most works in the related literature, which base the evaluation of the model on an intra-dataset validation. Thus, for the test of the classifier, they simply take a subset of samples generated by the same subjects and the same types of movements used for training. In our case, the final testbed with volunteers is completely independent from the one used for the training dataset (SisFall), which reveals the model’s ability to extrapolate its learning.

In any case, since both the SisFall training data and the tests conducted involved simulated falls performed on a cushioned surface, they may not fully represent the variability of real-world falls in different conditions. In this sense, the use of simulated falls to assess FDSs is highly controversial and is being increasingly challenged. A fall is an inherently unplanned, unstructured, and involuntary event, which leads to highly variable and complex dynamics as the person who falls performs compensatory movements to minimize injury. These compensatory actions, which help reduce the severity of the impact against the floor, are not present in ‘fake’ falls, which are mimicked by trained participants (normally healthy and young volunteers) who have been instructed on how to fall and who follow a specific set of repetitive patterns. In fact, some studies have indicated that the dynamics of real falls may more closely resemble other movements than those of fake falls [72,73]. Future studies should validate the model in more realistic scenarios to assess its effectiveness in uncontrolled situations.

## 6. Conclusions

This study presents a hybrid fall-detection system that combines a threshold-based model (TBM) with a CNN–LSTM algorithm, leveraging LoRa technology for low-power, long-range communication. The system achieves high accuracy (98.51%), sensitivity (96.70%), and specificity (99.44%), with an operational autonomy of 178 h in continuous monitoring. By integrating threshold-based detection and advanced classification, false alarms are minimized, and energy consumption is optimized, ensuring that data transmission occurs only when a potential fall is detected.

The transmission experiments showed a 96.67% detection rate for simulated falls and 100% accuracy in classifying daily activities as non-falls. LoRa communication proved reliable in various environments, achieving 100% packet reception in line-of-sight conditions for a 4-s data window (80 samples at 20 Hz).

These results highlight the robustness and scalability of the system, with applications in monitoring elderly individuals and people with reduced mobility, in application scenarios of continuous monitoring where minimizing false alarms and ensuring high accuracy is crucial for improving quality of life and safety.

However, since both the training data and real-time tests involved simulated falls, future research should validate the system in more dynamic scenarios and with real falls to improve its applicability and reliability in uncontrolled environments. Overall, this hybrid approach offers an efficient and accurate solution, balancing energy consumption, connectivity, and fall-detection performance, contributing to user safety and quality of life.

## 7. Limitations and Future Recommendations

- While the proposed system showed promising results in detecting falls based on acceleration peaks, it was validated under controlled indoor conditions and scenarios in which subjects typically fall on a hard surface. Future research should explore the system’s behavior under alternative conditions, such as soft surfaces (e.g., grass, mattresses) or falls caused by fainting, where acceleration values may be significantly lower.
- The Arduino Nano 33 BLE Sense general purpose board was used as a development tool for quick prototyping, aiming at leveraging its integrated IMU. However, unused components (e.g., MP34DT05, LPS22HB, HS3003) of the module, even when deactivated for our fall-detection application, may contribute to unnecessary power consumption (in the range of 1–5 μA). Future implementations should adopt a custom low-power embedded design that includes only the IMU.
- Although the system demonstrated reliable performance during real-time operation and 24-h continuous monitoring, future studies should include multi-week deployments to evaluate long-term stability, hardware durability, and operational reliability under real-world conditions.
- Informal feedback from five users indicated positive impressions regarding comfort and wearability, but no formal usability study was conducted. Future work should include structured user-centered evaluations to assess ergonomics and long-term comfort of the device, which is a critical aspect (normally neglected by the related literature) for the practical acceptance of this type of monitoring tool.
- We also recommend that future studies expand the number of participants and evaluated scenarios, incorporating greater user diversity and real-life conditions to enhance the system’s generalizability and applicability in broader contexts.

## Figures and Tables

**Table 1:** Overview of Fall Detection Studies Utilizing LPWAN Technologies.

Ref.	LPWAN Technology *	Sensor **	Algorithm Type	Accuracy	Sensitivity	Specificity	Battery Life	Transceiver
Escriba et al. [34]	Sigfox	Acc	-	-	-	-	3 days (low-power)/13 h (GPS), 30 mAh	N/A
Patel et al. [25]	LoRaWAN	Acc	Thresholding policies	96.93%	100%	94.25%	-	-
Valach et al. [26]	LoRaWAN	Acc	-	-	-	-	-	RFM95W
Manatarinat et al. [33]	NB-IoT	Acc & Gyr	-	-	-	-	-	-
Pena Queralta et al. [15]	LoRa	Acc, Gyr, Mag	LSTM	91.90%	95.3%	-	-	-
Scheurer et al. [27]	LoRa	Acc	-	-	-	-	-	EM9209
Cai et al. [16]	NB-IoT	Acc & Gyr	GBDT (acceleration dataset)	89.2%	-	-	-	-
Chang et al. [11]	LoRa	Acc, Gyr and IR (Infrared)	Thresholding policies	98.3%	-	-	-	-
Huynh et al. [10]	LoRa	Acc, Gyr, Mag	Thresholding policies	N/A	96.3%	96.2%	1 week–1 month	-
Lachtar et al. [28]	LoRa	Acc, Gyr, Mag	-	-	-	-	-	RFM95/96/97/98(W)
Zanaj et al. [29]	LoRaWAN	Acc, Gyr, Mag	-	-	-	-	23 h (500 mA/h), 36 h (800 mA/h)	SX1257
Liu et al. [14]	NB-IoT	Acc, Gyr, Mag	CNN	98.85%	98.86%	99.84%	-	-
Fan et al. [37]	NB-IoT	Acc & Gyr	-	-	-	-	-	M5310A
Li et al. [12]	LoRa	Acc & Gyr	Thresholding policies	85%	-	-	-	N/A
Qian et al. [7]	NB-IoT	Acc & Gyr	Thresholding policies	94.88%	95.25%	94.5%	-	BC-95
Salah et al. [9]	LoRa	Acc	K-NN (15 neighbors)	78.64%	81.07%	76.57%	More than 53 h (2000 mAh)	RFM95W
			K-NN (5 neighbors)	79.11%	80.06%	78.21%		
			CNN	95.55%	95.1%	94.86		
			LSTM	96.78%	97.87%	95.21%		
			SVM	82.27%	87.21%	78.48%		
Wong et al. [30]	LoRa	Acc, Gyr, Mag	-	-	-	-	-	SX1278 RA-02
Wu et al. [13]	NB-IoT	Acc & Gyr	Thresholding policies	90.1%	-	-	-	-
			GRU	92.9%	-	-		
Pierleoni et al. [38]	NB-IoT	Acc, Gyr, Mag	-	-	-	-	-	nRF9160

Notes: * LPWAN technologies include NB-IoT, LoRa, LoRaWAN, and Sigfox, which support long-range, low-power wireless communication. ** Sensors are abbreviated as follows: Gyr. (Gyroscope), Mag. (Magnetometer), Acc. (Accelerometer), and IR (Infrared sensor). Some parameters, such as orientation, can be inferred from inertial sensor data and are commonly used in FDSs. A dash (“-”) indicates that the corresponding reference does not explicitly provide this information.

**Table 2:** Equations for LSTM Layer Gates and Memory Update.

Component	Equation	Equation Number
Forget Gate	$G_{t}=\sigma \left(W_{G}\cdot \left[R_{t-1},Z_{t}\right]+B_{G}\right)$	(3)
Input Gate	$I_{t}=\sigma \left(W_{I}\cdot \left[R_{t-1},Z_{t}\right]+B_{I}\right)$	(4)
Cell Candidate	$\tilde{S_{t}}=\tanh\left(W_{S}\cdot \left[R_{t-1},Z_{t}\right]+B_{S}\right)$	(5)
Memory Update	$S_{t}=G_{t}\times S_{t-1}+I_{t}\times \tilde{S_{t}}$	(6)
Output Gate	$O_{t}=\sigma \left(W_{O}\cdot \left[R_{t-1},Z_{t}\right]+B_{O}\right)$	(7)
Output	$R_{t}=O_{t}\times \tanh\left(S_{t}\right)$	(8)

where σ represents the logistic activation function, and tanh denotes the hyperbolic tangent function. The variable $R_{t}$ defines the hidden state at time t, while $S_{t}$ corresponds to the memory cell state at the same time step. Additionally, $Z_{t}$ represents the input at time *t* whereas W and B are the weight matrices and bias terms, respectively.

**Table 3:** Comparison of model performance.

Model	Accuracy (%)	Sensitivity (%)	Specificity (%)
K-NN (5 neighbors)	92.57	91.21	93.26
K-NN (15 neighbors)	92.19	91.21	92.70
SVM	91.82	93.41	91.01
CNN	95.54	91.21	97.75
LSTM (with Batch Norm.)	93.31	87.91	96.07
CNN–LSTM	98.51	96.70	99.44

**Table 4:** Energy consumption (mean current drain) for different operating states.

Operating State	Current (mA)	Description
Microcontroller active with LoRa module active	24.3	The microcontroller processes data, and the LoRa module is active without data transmission.
Microcontroller active with LoRa module suspended	8.2	The microcontroller captures accelerometer data, while the LoRa module remains suspended.
Microcontroller in sleep mode with LoRa module suspended	4.5	The microcontroller operates in low-power mode, waking up only to capture data at 20 Hz.
Data transmission	56.2	The LoRa module transmits 4-s data windows.

**Table 5:** Comparative Evaluation of Model Performance.

Test Scenario	Distance (m)	PRR (%)	SNR (dB)	RSSI (dBm)	Obstacles	Gateway Configuration
Indoor (line-of-sight)	15	100.00	14	−49	None	Gateway on the same floor
Indoor (non-line-of-sight)	50	95.00	8	−88	five hollow brick walls	Gateway on the same floor
Indoor (from an upper floor)	10	98.75	12	−75	one floor (concrete slab)	Gateway on the lower floor
Suburban (line-of-sight)	360	100.00	−2	−82	None	Gateway at 40 m altitude
Urban	750	87.00	−6	−91	Buildings and trees	Gateway on the 13th floor of a building

**Table 6:** Results for Fall Movements.

Description of the Simulated Falls	Number of Falls	Number of Falls Presumed by Thresholding	Number of Falls Identified by the CNN-LSTM Model	Global Sensitivity (%)
Forward fall ending in a lying position	15	15	15	100.00%
Lateral fall	15	15	14	93.33%
Backward fall with rotation, ending face-down	15	15	14	93.33%
Backward fall ending in a supine position	15	15	15	100.00%
Total	60	60	58	96.67%

**Table 7:** Results for Activities of Daily Living (ADLs).

Description	Number of Movements	Movements Ignored by Thresholding	Classified as Non-Fall by CNN-LSTM	Specificity (%)
Lying down and getting up	15	14	1/1	100.00%
Sitting in a chair and standing up again	15	14	1/1	100.00%
Walking	15	15	0/0	100.00%
Bending down and standing up	15	13	2/2	100.00%

**Table 8:** Cost breakdown of the wearable fall detection system.

Component	Description	Quantity	Unit Cost (USD) *	Total (USD)
Arduino Nano 33 BLE Rev2	Main microcontroller unit with integrated BLE	1	$29	$29
Reyax RYLR998	LoRa transceiver module	2	$13	$26
ESP32 DevKitC	Gateway microcontroller with Wi-Fi	1	$10	$10
Custom PCB via JLCPCB	Fabricated double-layer PCB board	1	$5	$5
3D-Printed Enclosure + TPU	PLA enclosure with flexible rubber case	1	$7	$7
Battery + Connectors	Li-Po battery, wiring, pin headers	1	$6	$6
Hardware Subtotal	Total for wearable and gateway hardware	—	—	$83
AWS EC2 t2.micro (monthly)	Cloud instance for model inference/storage	1 month	$8.35	$8.35
Total Estimated Cost	Includes hardware and one-month cloud service	—	—	$91.35

Note: * Prices retrieved on 4 May 2025, from official stores and online commercial platforms.

## Data Availability

The original contributions presented in the study are included in the article, further inquiries can be directed to the corresponding authors.
